# A small library of chalcones induce liver cancer cell death through Akt phosphorylation inhibition

**DOI:** 10.1038/s41598-020-68775-9

**Published:** 2020-07-16

**Authors:** Irem Durmaz Sahin, Michael S. Christodoulou, Ece Akhan Guzelcan, Altay Koyas, Cigdem Karaca, Daniele Passarella, Rengul Cetin-Atalay

**Affiliations:** 1 School of Medicine, Koc University, 34450 Istanbul, Turkey; 2 DISFARM, Sezione di Chimica Generale e Organica “A. Marchesini” Universitádegli Studi di Milano, via Venezian 21, 20133 Milano, Italy; 3 CanSyL, Graduate School of Informatics, Middle East Technical University, 06800 Ankara, Turkey; 4 Faculty of Dentistry, Department of Oral and Maxillofacial Surgery, Hacettepe University, 06230 Ankara, Turkey; 5 Department of Chemistry, Universitá degli Studi di Milano, Via Golgi 19, 20133 Milano, Italy

**Keywords:** Drug development, Hepatocellular carcinoma, Small molecules

## Abstract

Hepatocellular carcinoma (HCC) ranks as the fifth most common and the second deadliest cancer worldwide. HCC is extremely resistant to the conventional chemotherapeutics. Hence, it is vital to develop new treatment options. Chalcones were previously shown to have anticancer activities in other cancer types. In this study, 11 chalcones along with quercetin, papaverin, catechin, Sorafenib and 5FU were analyzed for their bioactivities on 6 HCC cell lines and on dental pulp stem cells (DPSC) which differentiates into hepatocytes, and is used as a model for untransformed control cells. 3 of the chalcones (**1**, **9** and **11**) were selected for further investigation due to their high cytotoxicity against liver cancer cells and compared to the other clinically established compounds. Chalcones did not show significant bioactivity ($\hbox {IC}_{50}>20\upmu \hbox {M}$) on dental pulp stem cells. Cell cycle analysis revealed that these 3 chalcone-molecules induced SubG1/G1 arrest. Akt protein phosphorylation was inhibited by these molecules in PTEN deficient, drug resistant, mesenchymal like Mahlavu cells leading to the activation of p21 and the inhibition of NF$\kappa$B-p65 transcription factor. Hence the chalcones induced apoptotic cell death pathway through NF$\kappa$B-p65 inhibition. On the other hand, these molecules triggered p21 dependent activation of Rb protein and thereby inhibition of cell cycle and cell growth in liver cancer cells. Involvement of PI3K/Akt pathway hyperactivation was previously described in survival of liver cancer cells as carcinogenic event. Therefore, our results indicated that these chalcones can be considered as candidates for liver cancer therapeutics particularly when PI3K/Akt pathway involved in tumor development.

## Introduction

Hepatocellular carcinoma (HCC) ranks as the fifth most common and the second deadliest cancer type worldwide^1^. The prognosis of HCC requires a multi-step process starting with chronic liver disease progressing through formation of dysplastic nodules and resulting in liver carcinogenesis upon gathering of different genomic alterations^2^. HCC is extremely resistant to conventional chemo- and radio-therapies^3^. The FDA (US-Food and Drug Administration) agency approved targeted drug for advanced HCC is Sorafenib which can only prolong survival 2–5 months^4^. Thus it is crucial to develop new treatment or prevention strategies for HCC. Sorafenib, which is a multikinase inhibitor acts trough RAF/MEK/ERK pathway and VEGFR/PDGFR tyrosine kinases. Therefore, alternative signaling pathways involved in liver cancer cell survival can be targeted in liver cancer therapy^5^. PI3K/Akt pathway was shown to be hyperactive in liver cancer cells which can be exploited for this purpose^6^. PI3K/Akt pathway is critical in the regulation of cell proliferation, cell survival and angiogenesis^7–9^. The lipid phosphatase and tensin homolog (PTEN) negatively regulates the activation of Akt by PI3K. The loss of PTEN tumor suppressor leads to the constitutive activation of Akt by PI3K, hence leading to the activation of cell survival and growth. PTEN deletion is frequently observed in HCC^10^. Therefore this pathway plays a central role in the development of several cancers including HCC^8, 11, 12^. The deletion of PTEN, activation mutation of PI3K or other receptor tyrosine kinases may result in deregulation of PI3K/Akt pathway in HCC. Especially PTEN was previously shown to be deleted in Mahlavu cells, which is mesenchymal-like drug resistant poor differentiated aggressive HCC cell line^13^.

Chalcones are a group of natural compounds that are widely found in the plant kingdom^14^. Their structure is presented in a variety of biologically active molecules including synthetic and natural products and are considered as open chain intermediates in the synthesis of flavones^15^. Chalcones possess a wide range of biological properties such as anti-viral, anti-oxidant, anti-fungal, anti-inflammatory, anti-microbial, anti-HIV and anticancer activities^16, 17^. The presence of the $\alpha$,$\beta$-unsaturated carbonyl motif is believed to be responsible for their biological activities since chalcones act as Michael acceptors by trapping thiols in a biological media^18^. Different pathways have been elucidated in which chalcones induce their activity. Therefore, chalcones can be used as inhibitors of the multi-drug resistance (MDR) Channels, the hormonal Milieu, histone deacetylases (HDAC), the p53 degradation, the JAK/STAT signaling pathway, angiogenesis, and cellular proliferation. Furthermore, chalcones are exploited as cytotoxic agents^19^.

In this study, following our interest in the synthesis and the identification of compounds with anticancer related bioactivities^20–32^, eleven chalcones were synthesized and tested against HCC cell lines and dental pulp stem cells which are reported to be hepatic progenitor properties^33^. DPSCs can proliferate and differentiate rapidly into various lineages including hepatocytes therefore they present a good model for untransformed control cells^34^. We documented that three of the molecules were highly cytotoxic toward poorly differentiated aggressive liver cancer cells. Treatment with these molecules resulted in SubG1/G1 cell cycle arrest induced apoptosis through deregulation of Akt related pathways.

## Results

### Chemistry

#### General procedure for the preparation of chalcones

**Figure 1:**
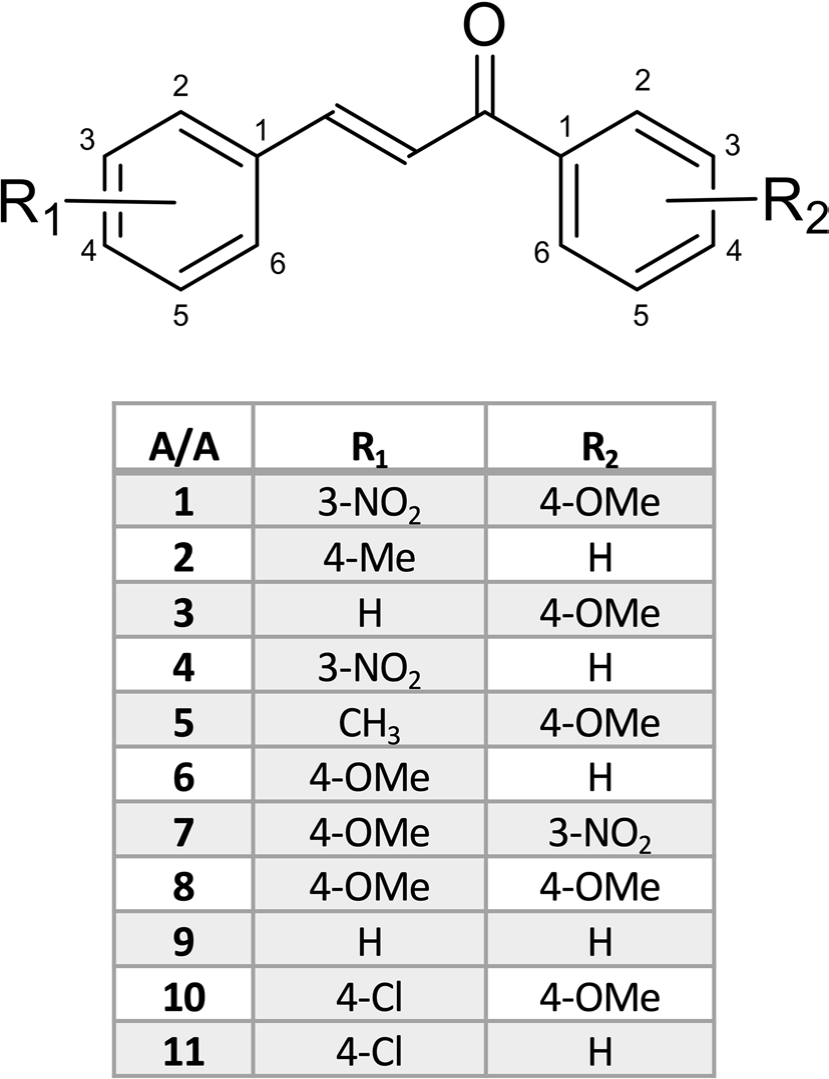
Structures of the synthesized chalcones.

An aqueous solution of sodium hydroxide (30%, 25 mL) was slowly added to a methanol solution (30 mL) of the appropriate acetophenone (5.0 mmol). After the solution had been cooled to room temperature, the appropriate benzaldehyde (6.0 mmol) was added. The mixture was stirred at room temperature overnight and was then poured into water (100 mL). The obtained solid was filtered, washed with water until neutral pH and recrystallized from ethanol. The synthesized chalcones are presented in Fig. 1.

### Pharmacology

#### Cytotoxic evaluation of the synthesized compounds

The bioactivities of the synthesized chalcones were assessed on human liver cancer cell lines and on DPSCs with NCI-SRB assay in vitro. DPSCs used to asses the bioactivity of chalcones on untransformed control cells with the potential of hepatic lineage^34^. The cells were treated with the range of increasing concentrations of the compounds (2.5–40 μM) for 72 h. We also included 5-Fluorouracil which is used in clinics for gastrointestinal cancers and Sorafenib. All results were normalized to DMSO negative control measurements. The experiment was performed in triplicate. Results revealed that especially compounds **1**, **2**, **5**, **6**, **9** and **11** possess high bioactivities in liver cancer cells (Table 1). Compounds **1**, **9** and **11** were chosen for further analysis due to their higher bioactivities on aggressive, poorly-differentiated liver cancer cell lines (Mahlavu, FOCUS, SNU475) compared to the other chalcones tested in this study (Table 1).

**Table 1:** IC_50_ ($\upmu \hbox {M}$) values of the compounds.

	Huh7	HepG2	Hep3B	Mahlavu	FOCUS	SNU475	DPSC
1	1.3	4.2	6.2	2.2	4.9	3	NI
2	4.1	5.8	5.6	5.1	11.4	9.8	28.7
3	6.4	7.6	18	9.4	20	13.1	NI
4	44.4	51.6	NI*	24.8	NI	23.8	NI
5	3	9.1	8.5	9.2	5.4	14.5	NI
6	6.4	7.6	7.8	6.3	6.5	10	NI
7	44.9	58.9	47.5	31.1	NI	NI	NI
8	17.9	26	22.1	17.3	21.5	21.6	NI
9	5.3	7.3	7.3	6.3	3.3	5	25.7
10	11.9	13.9	16.5	14.6	15.9	20	NI
11	4.3	6.5	5.9	5.3	3.1	9	19.1
Quercetin	12.5	8.4	21.8	11.3	17.9	11.6	21.2
Papaverine hydrochloride	8.5	4.9	20.4	52.3	1.7	17.8	NI
Catechin hydrate	NI	NI	NI	NI	NI	NI	NI
Sorafenib	1.3	5.6	NA	7.9	NA	7.5	7.1
5-Fluorouracil	30.7	5	15.2	10	7.7	NA	23.7

*DPSC* dental pulp stem cells, *NI* no Inhibition (*IC*_50_ > 40 (μM)), *NA* not applicable, $R^2 >0.8$.

**Figure 2:**
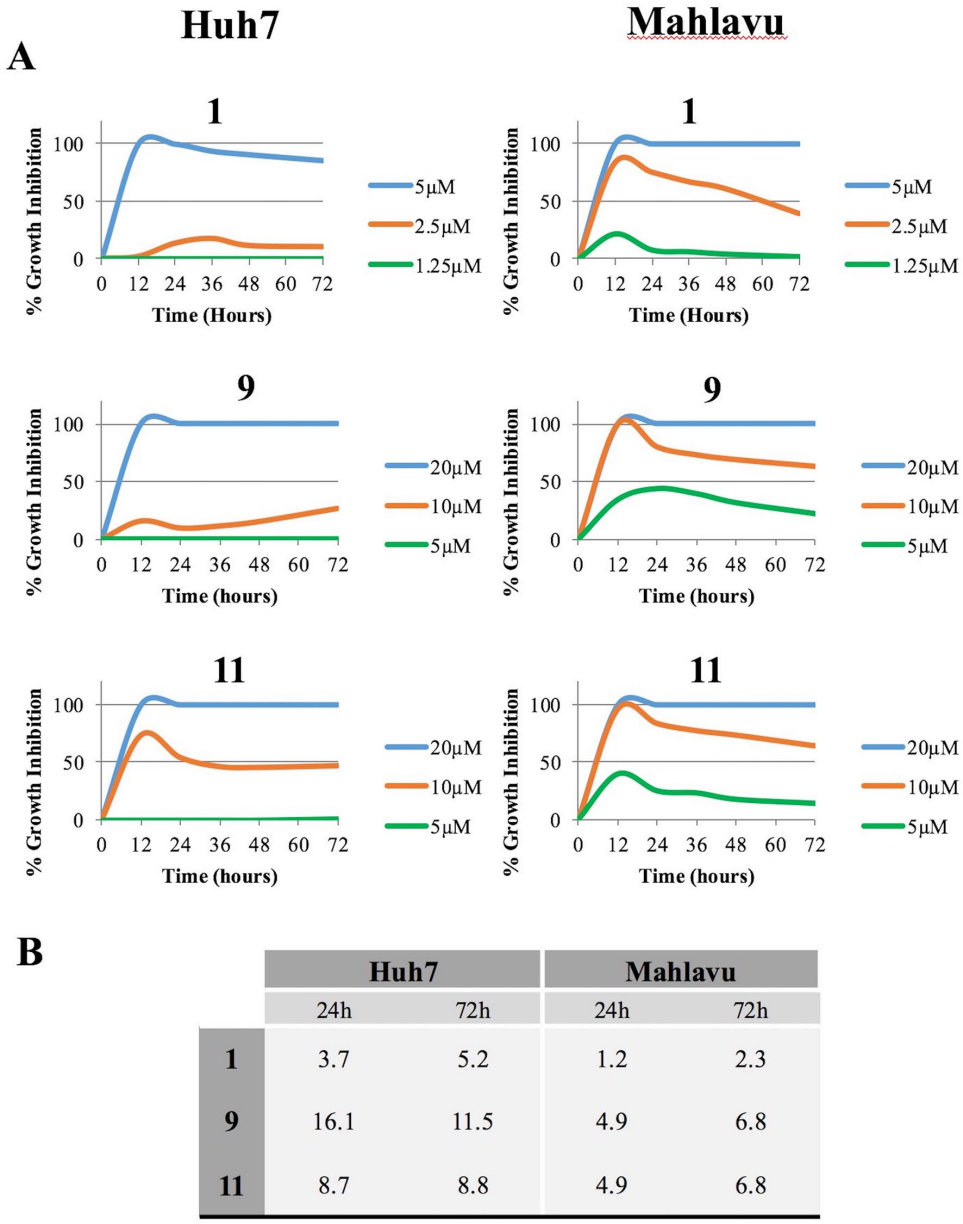
Real-time cell growth analysis. Human liver cancer cells Huh7 (left panel) and Mahlavu (right panel) were treated with the selected compounds. Cell index measurements were obtained by RT-CES software. DMSO was used as negative control. (**A**) The growth inhibition values of the compounds were obtained by the normalization with DMSO. Experiments were performed in triplicate. (**B**) IC_50_ values calculated from RT-CES experiment data.

The selected 3 compounds were further analyzed with real-time cell electronic sensing (RT-CES) system to assess the real-time growth behavior of the cells treated with the compounds. Huh7 and Mahlavu liver cancer cells were treated with the selected chalcones and monitored with RT-CES. Results of the compounds were normalized to data of negative control DMSO. RT-CES experiment suggested that the compounds were more effective in the first 12–24 h of the treatment, then the bioactivities of the compounds stayed stable (Fig. 2).

#### Characterization of the cell death induced by chalcones

**Figure 3:**
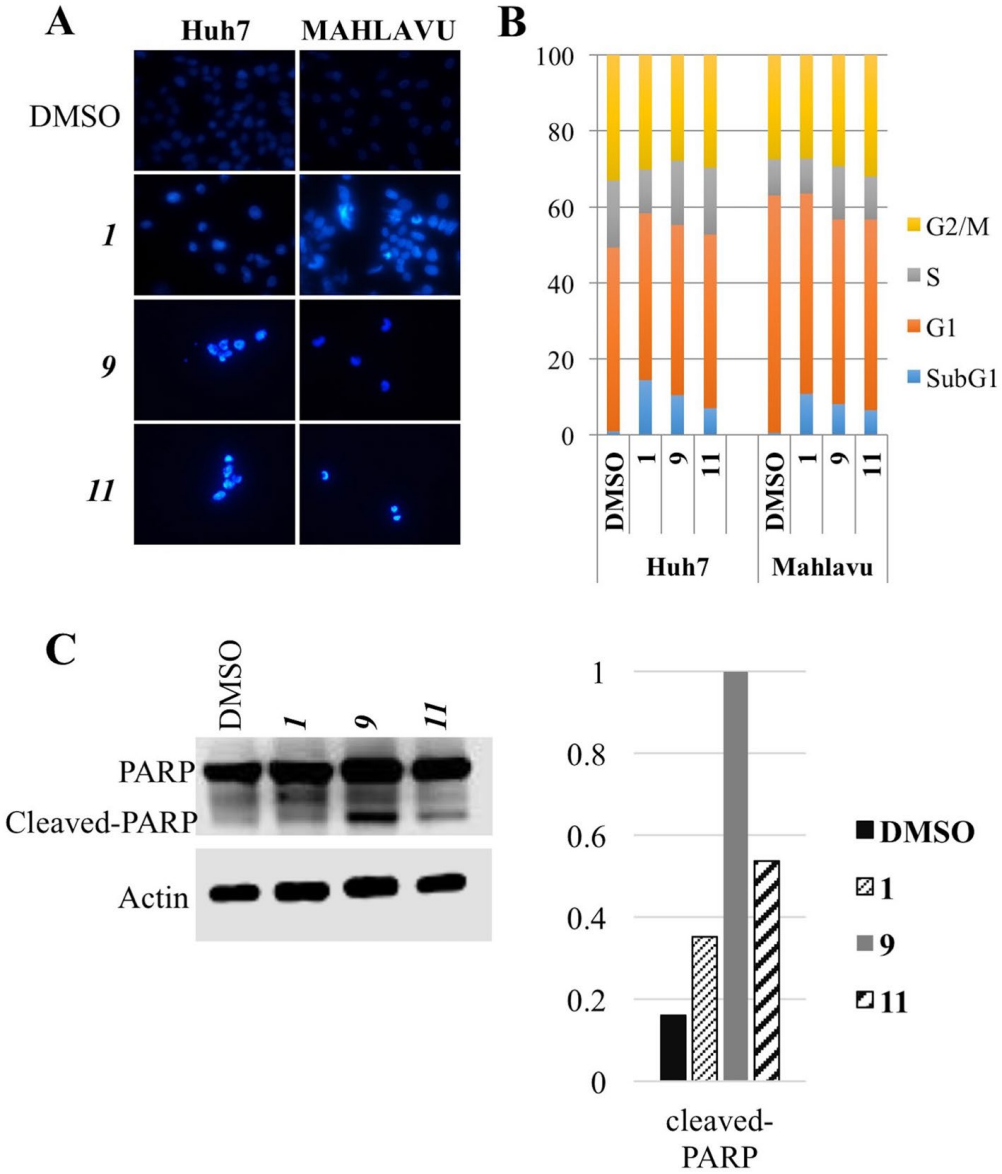
Cell death induced by the chalcones. (**A**) Nuclear staining of the liver cancer cells treated with the chalcones. Human liver cancer cells (Huh7 and Mahlavu) were treated with the IC_50_ concentrations of the chalcones **1**, **9**, **11**. After 72 h of incubation with the compounds or DMSO control, Hoechst (33258) staining was performed. Images were taken with fluorescent microscope (×40). Due to the strong cytotoxic activities of the chalcones the number of the cells in objective area was much less than DMSO controls. (**B**) Cell cycle distribution of liver cancer cells. Huh7 and Mahlavu cells were treated for 72 h with IC_50_ concentrations of the compounds or DMSO control. SubG1 cell cycle arrest was observed upon treatment with the compounds (blue). (**C**) Investigation of PARP cleavage in Huh7 and Mahlavu cells treated with the selected chalcones for 24 h.

Human liver cancer cells Mahlavu and Huh7 were treated with the compounds according to the IC_50_ values obtained from RT-CES experiments or DMSO controls for 72hr. Hoechst-dye-stained cells were observed under fluorescent microscope revealing distinctive morphologies comparing to DMSO controls. Nuclear condensation and horse-shoe like structures suggests apoptosis induction which need to be further confirmed with detailed experiments (Fig. 3A). We further assessed the effects of the selected chalcones **1**, **9**, and **11** on the cell cycle progression analysis of liver cancer cells. Human liver cancer cells Mahlavu and Huh7 were treated with the compounds according to the IC_50_ values obtained from RT-CES for 72 h. Then the DNA in the treated cells were stained with propidium iodide and analyzed with fluorescent-activated cell sorter. Results showed that the chalcones induced SubG1 cell cycle arrest in human liver cancer cell lines (Fig. 3B). The type of cell death induced by the compounds was suggested to be apoptosis with the results of Hoechst nuclear staining and cell cycle analysis. In order to confirm the induction of apoptosis in human liver cancer cells treated with the chalcones, cleavage of PARP protein (marker of apoptosis) was investigated. Western blot results indicated that PARP cleavage was present significantly in cells treated with **9** and **11** (Fig. 3C). These results confirmed that the compounds induced apoptotic cell death in liver cancer cells.

**Figure 4:**
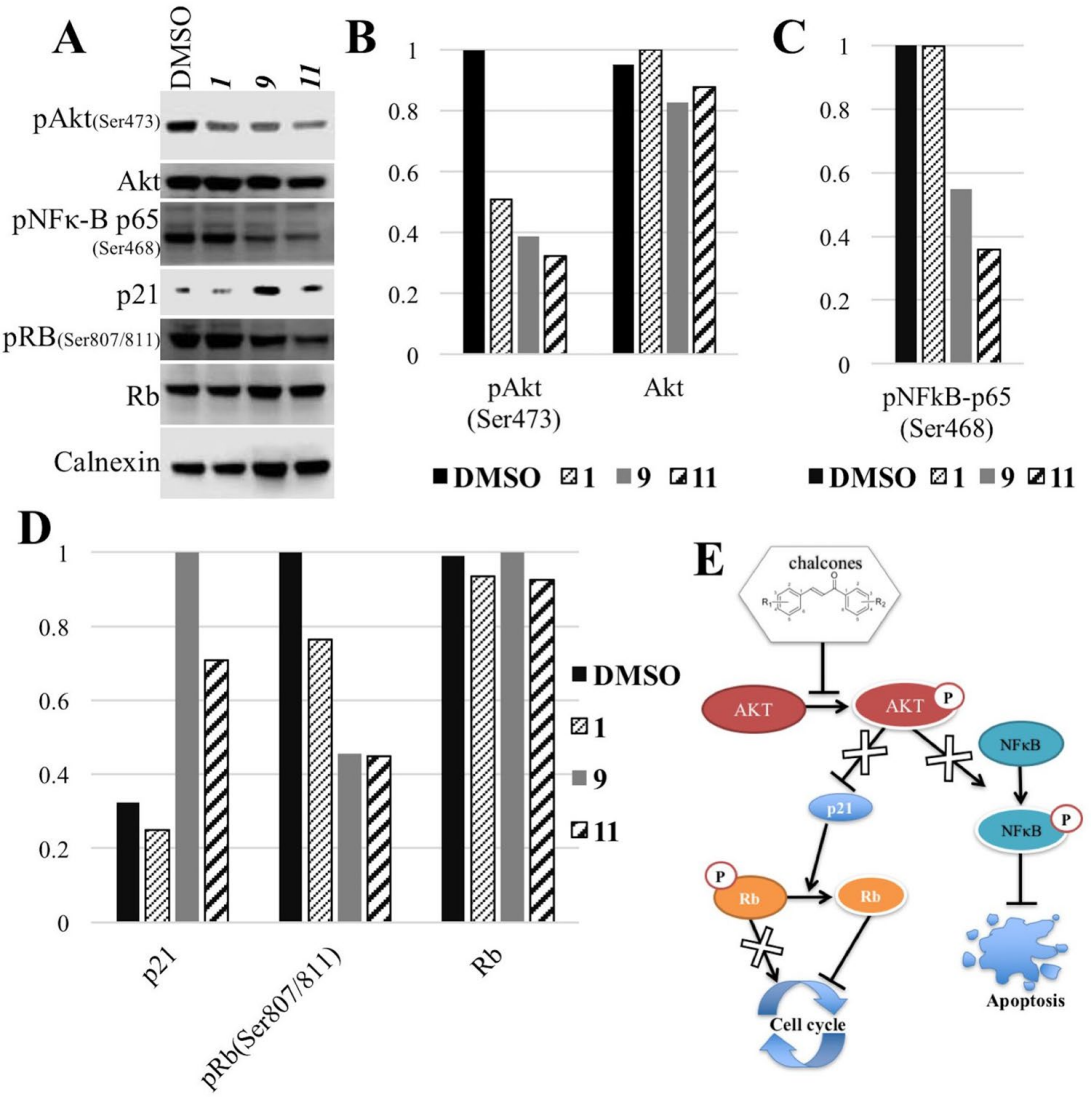
Proteins targeted by the compounds. Human liver cancer cells (Mahlavu) were treated with the IC_50_ concentrations of the selected compounds or DMSO control for 24 h. (**A**) Western blot analysis showed that Akt protein is inhibited in liver cancer cells treated with the compounds resulting in alterations of p21 and NFkB proteins. (**B**–**D**) Quantification of the results using ImageJ software. (**E**) Schematic representation of the molecular mechanism of action of the compounds. Blocked signaling is crossed.

#### Cellular pathway components targeted by the chalcones 1, 9, 11

In order to investigate the significant bioactivity of the chalcones **1**, **9** and **11** on human liver cancer cell line Mahlavu which has an hyperactive PI3K/Akt pathway due to PTEN-deficiency^13^, we analyzed the effect of the chalcones on the active phosphorylated Akt levels in this cell line. Western blot analysis revealed that treatment with all three compounds resulted in decrease in the levels of pAkt upon treatment for 24 h with IC_50_ values given in Fig. 2 (Fig. 4A,B). The decrease in the Akt protein phosphorylation suggested that the chalcones interfere with Akt protein activation in Mahlavu cells (Fig. 4A,B) which is more pronounced in the treatments with **9** and **11**.

In the literature, it was shown that the anti-cancer effect of chalcones on uterus leiomyoma cells was through an increase in p21 protein^35^. Thus we also examined the levels of p21 in our cells. Furthermore, p21 is also a downstream element of Akt pathway. The results presented in Fig. 4A,C indicated that, the p21 protein levels were increased significantly in cells treated with chalcones **9** and **11**, **9**, being the most significant (Fig. 4). Cell cycle analysis revealed a cell cycle arrest induced by the compounds (Fig. 3B). Therefore, the effect on Rb protein, which is also a downstream element of p21, was investigated in the presence of the chalcones **1**, **9** and **11**. The phosphorylated Rb protein levels were decreased significantly in the cells treated with **9** and **11** (Fig. 4). This suggested that, treatment with the compounds resulted in activation of Rb through its dephosphorylation (Fig. 4A,C) inducing cell cycle arrest (Fig. 3B).

NFB is a transcription factor which is a fundamental component of inflammation pathway. This protein has also crucial roles in cell proliferation and apoptosis, thus cancer progression^36^. In addition, previous studies showed that cardamonin analogs (type of chalcones) suppress NFB pathway in lung cancer cells^37^. Since the inflammation is one of the hallmarks of cancer, we also investigated the effect of our compounds on NFB protein. The phosphorylation of NFB-p65 at Ser468 decreased significantly upon treatment with **9** and **11** (Fig. 4A,D). This form of NFB is the activated form which translocates into the nucleus to function as a transcription factor. Decrease in this phosphorylated form of the protein suggested that NFB-p65 is altered upon treatment with the chalcones **9** and **11** (Fig. 4A,D).

## Discussion

In this study, recently synthesized chalcones were the first time examined for their anti-proliferative properties in cancer cells and compared with 5-FU and Sorafenib. 5-FU is a chemotherapeutic agent frequently used in gastrointestinal cancers and Sorafenib is the first FDA (US-Food and Drug Administration) agency approved drug in primary liver cancer. Most of the compounds had moderate to significant bioactivities on liver cancer cells tested (Table 1). Furthermore drugs had no significant cytotoxic activity on dental pulp stem cells which we used a model for untransformed hepatic progenitor cells. 3 of the compounds (**1**, **9** and **11**) were selected for further analysis according to their bioactivities on poorly differentiated aggressive liver cancer cells (Table 1 and Fig. 2). The cell death analysis with the chalcones **1**, **9** and **11** revealed that these three molecules induce SubG1 cell cycle arrest and apoptosis (Fig. 3). Previously it was shown that several different pathways can be targeted with chalcones in various cancers. In this study, PTEN-deficient, Akt hyperactive aggressive liver cancer cell line Mahlavu cells were treated with the newly synthesized chalcones and molecular mechanism of action were analyzed. Treatment with the compounds induced decrease in pAkt levels suggesting induction of an inhibition of Akt protein (Fig. 3). Moreover, decrease in phosphorylated NFB-p65 (Ser468) and increase in p21 protein were observed in treated cells (Fig. 4). Akt protein was previously shown be one of the regulators of the activities of these two proteins^38, 39^. Thus, alteration of Akt protein activity by the chalchones result in the activation of p21 protein along with the inhibition of NFB-p65 protein phosphorylation. NFB-p65 is a transcription factor which is activated by phosphorylation and then translocate into nucleus^36, 40^. Our compounds causes decrease in its phosphorylation and thereby its activity. Active NFB-p65 was shown to have fundamental role in cancer progression by inhibition of apoptosis^36, 41^. Thus, the alteration in phosphorilation of NFB-p65 by the chalcones **9** and **11** results in induction of apoptosis in liver cancer cells (Fig. 4). Moreover, in the cells treated with the compounds, phosphorylation of Rb protein, which is a cell cycle regulator, was decreased. Under normal conditions, during cell division Rb protein is inhibited by phosphorylation. Decrease in the phosphorylation of Rb, meaning its activation, results in cell cycle arrest in human liver cancer cells treated with the chalchones **1**, **9** and **11** (Fig. 4). In this study our results indicates that these newly synthesized chalcones can be considered as good candidates for liver cancer therapeutics particularly chalcone **9**. For future perspectives, the design of new class of compounds with this scaffold will be studied with the aim to improve the *in vitro* performance and analyze the activity of the compounds in vivo on animal models.

## Methods

### Cell culture

Well differentiated human primary liver cancer cell lines Huh7, HepG2 and Hep3B, and poorly differentiated Mahlavu, FOCUS and SNU475 HCC cells were cultured in Dulbecco’s Modified Eagle’s Standard (DMEM) medium supplemented with 10% Fetal Bovine Serum (FBS), 100 units/mL penicillin and 100 lg/mL streptomycin (Gibco, Invitrogen, Carlsbad, CA, USA). 0.1 mM nonessential amino acids (NEAA) which are specific to HCC cell lines also added to culture media. Cells were cultured in a 5% CO2 incubator at $37\,^\circ \hbox {C}$

### Dental pulp stem cell isolation

Dental pulp stem cells (DPSCs) were isolated from anonymised unidentified healthy intact wisdom tooth. DPSCs were isolated within few hours upon wisdom teeth surgery from patients above 18 years old. All were informed about the procedures, and their consents were obtained. Teeth were broken carefully in order to reach the dental pulp area. After the surgical extraction, pulp tissues from maxillary and mandibular teeth were washed several times with ice-cold PBS (Gibco, Cat: 14190-169) and transferred within 2 hours on ice into DMEM-F12 media (Gibco, Cat: 11320033) supplemented with 10% FBS (Gibco, Cat: 10270), 1× Penicillin & Streptomycin (Gibco, Cat:15140-122), 2,5 $\upmu$g/ml Amphotericin B (Biological Industries, Cat:03-028-1B) and 5 $\upmu$g/ml Plasmocin (Invivogen, Cat: ant-mpp). Pulp tissue was shredded by scalpel and chemically digested with Liberase (Merck, Cat: 5401089001) approx. 1 U/ml for 45 minutes at $37\,^\circ \hbox {C}$. Then, the cells were seeded onto flasks in 10ml DMEM-F12 media (Lonza) supplemented with 1% penicillin/streptomycin solution (Hyclone) and 15% FBS (Fetal Bovine Serum,Hyclone, Logan, UT, USA) and cultured in a 5% CO2 incubator at $37\,^\circ \hbox {C}$. 10 day of culturing was usually optimal to obtain DPSCs.

### Dental pulp stem cell characterizaton with flow cytometry

Trypsinized (Biological Industries, Cat: BI03-052-1B) cells were collected and washed with ice-cold PBS once. Next, cells were fixed with 4% Paraformaldehyde (Sigma, Cat:158127) in PBS for 20 minutes at room temperature and centrifuged at 1500 rpm for 5 minutes; following by resuspension in stain buffer (BD, Cat: 554656) in a concentration scale of 1 × 106 cells/ml. Following antibodies were used as described; EpCAM-APC (BD, 347200) (1:100 v/v), CD133-PE (BioLegend, 372804) (1:100 v/v), CD44-FITC (Miltenyi Biotec, Cat: 130-095-195) (1:10 v/v) and CD90-FITC (Miltenyi Biotec, Cat: 130-095-403) (1:10 v/v). For unstained controls, IgG1 Isotypes; IgG1-FITC (Immunostep, Cat: ICIGG1F-100), IgG1-PE (Immunostep, Cat: ICIGG1PE-50), and IgG1-APC (BD, Cat: 555751); were used as 1:20 (v/v). For staining, cells were incubated with antibodies for 30 minutes in room temperature at dark and washed once with staining buffer. Stained cells were analyzed on NovoCyte Flow Cytometer System (Acea) and analysis was performed via NovoExpress Software (Supplementary Fig. S1).

### NCI-60 sulforhodamine B assay for in vitro cytotoxicity screening

Primary liver cancer cells Huh7, HepG2, Hep3B, Mahlavu, FOCUS, Snu475 along with hepatic progenitor Dental pulp stem cells were seeded into 96-well plates (1,000–3,000 HCC cell/well and 10,000 cells DPSC/well ) for 24 h. The cells were then treated with increasing concentrations of the chalcones ($2.5-40 \mu \hbox {M}$). DMSO (AppliChem Biochemica, Darmstadt, Germany) was used as negative control. The growth has stopped at the end of 72 h by fixing cold with 10% (v/v) trichloroacetic acid (Merck, Schuchardt, Germany). Cells were then stained with 0.4% (m/v) of sulforhodamine (Sigma-Aldrich, St. Louis, USA) in 1% acetic acid solution. The absorbency values were acquired at 515 nm. All experiments were done in triplicate.

### Real-time cell electronic sensing (RT-CES analysis)

Huh7 and Mahlavu cells were inoculated into the e-plate (1000–2000 cells/well). The attachment, spreading, and proliferation of the cells were monitored every 30 minutes using the Xcelligence^®^ Real-Time Cell Analysis system (ACEA Biosciences Inc.) in a cell culture incubator. The electronic readout (cell-sensor impedance) was displayed as an arbitrary unit called the cell index (CI). When cells reach to an cell index (CI) impedance values about 1.5 usually in 24 hours cells were treated with the chalcones **1**, **9** and **11**. DMSO was used as a negative control. Each experiment was repeated three times. The CI value was noted every 10 minutes for the first 24 hours and then every 30 minutes upon chalcone treatments. The cell inhibition rate calculated as follows $(\%) = [1 -(\hbox {CI}_{\mathrm{treatedcells}}/\hbox {CI}_{\mathrm{DMSO}})] \times 100$.

### Nuclear stain

Huh7 and Mahlavu cells were inoculated in 6-well plates for 24 h. The cells were treated with IC_50_ concentrations (Table 1) of the compounds (**1**, **9** and **11**) for 72 h. Hoescht 33258 (Sigma–Aldrich) staining was done to visualize the nuclear condensation. Cells were fixed with 1 mL of cold methanol and the samples were incubated with 3 mg/mL of Hoescht, and examined under fluorescent microscopy (40×).

### Cell cycle analysis

Cells were treated with IC_50_ concentrations of the chalcones (**1**, **9** and **11**)(Table 1) for 72 h. Then samples were stained with propidium iodide which binds to DNA and analyzed with MUSE^®^ Cell Cycle Assay Kit (EMD Milipore).

### Western blot analysis

Poorly differentiated liver cancer cell line Mahlavu cells were cultured in 100 mm culture dish for 24 h. Growth medium was then replaced with IC_50_ concentrations of chalcones **1**, **9** and **11** or DMSO (control) supplemented medium. cells were incubated for 24 hours then were scraped and collected for western blot analysis. Anti-PARP antibody (Cell Signaling, 9532), pAkt (Ser473) antibody (Cell Signaling, 9271), Akt antibody (Cell Signaling, 9272), pNFkB-p65 (Ser468) antibody (Snat Cruz, sc101750), p21 antibody (Millipore, 05345), pRb (Ser807/811) antibody (Cell Signaling, 9308), Rb antibody (Santa Cruz, sc102), actin antibody (Sigma, A5441) and calnexin antibody (Sigma, C4731) were used as primary antibodies. Anti-rabbit (6154) and anti-mouse (0168) secondary antibodies were used. ImageJ tool (^42^) used for protein band intensity comparison. Original Full length blots are given as Supplementary Information.

### Ethics statement

The study and use of dental pulp cells was approved by Hacettepe University (permit no 2019/06-49) and informed consent was obtained from all participants. (All patients were above 18 years old). The study was conducted according to the institutional guidelines and regulations.

## Supplementary information

Supplementray information

## Data Availability

All data generated or analysed during this study are included in this published article.
