# Supplementary material for: A small library of chalcones induce liver cancer cell death through Akt phosphorylation inhibition
Source: Sci Rep. 2020 Jul 16;10:11814. doi: 10.1038/s41598-020-68775-9 (PMC7367369; doi:10.1038/s41598-020-68775-9)
Supplement: Supplementary file 1 — Supplementray information [file 41598_2020_68775_MOESM1_ESM.pdf]

A small library of chalcones induce liver cancer cell death through Akt phosphorylation inhibition

Irem Durmaz Sahin, Michael S. Christodoulou, Ece Akhan Guzelcan, Altay Koyas, Cigdem Karaca, Daniele Passarella, and Rengul Cetin-Atalay

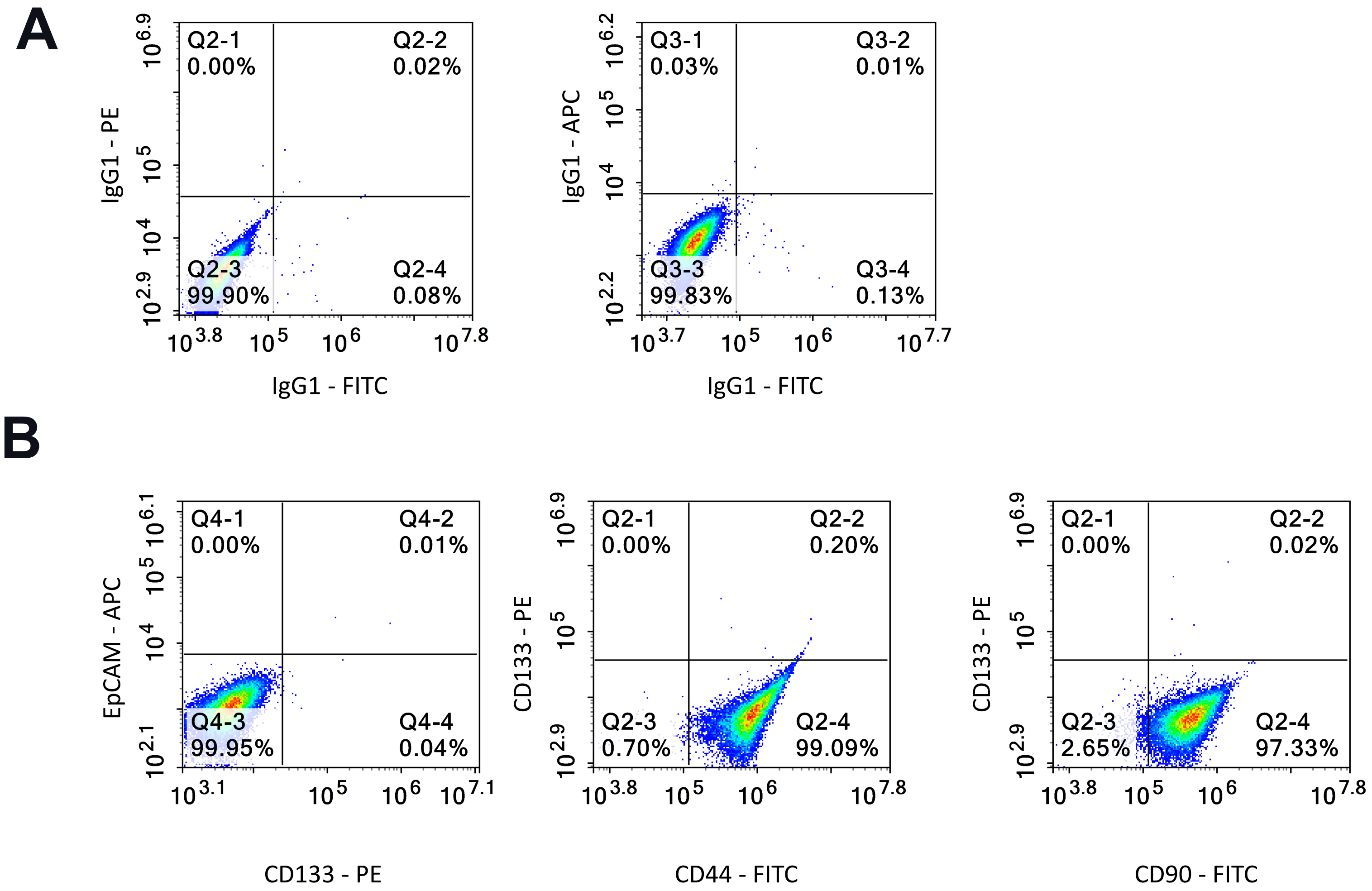

Supplementary Figure S1: Characterization of DPSCs by flow cytometry. Images demonstrate the immuphenotyping of **A** Unstained; **B** Stained cells for CD44, CD90, CD133 and EpCAM markers. Intensity values for each marker display the threshold for positivity based on the IgG1 control stainings. These resuts pointed out that the cell population was positive for CD44+ and CD90+ markers, which is correlated with the general marker expression panel of DPSCs. In conclusion, the isolated cell population displayed characteristic mesenchymal stem cell properties.

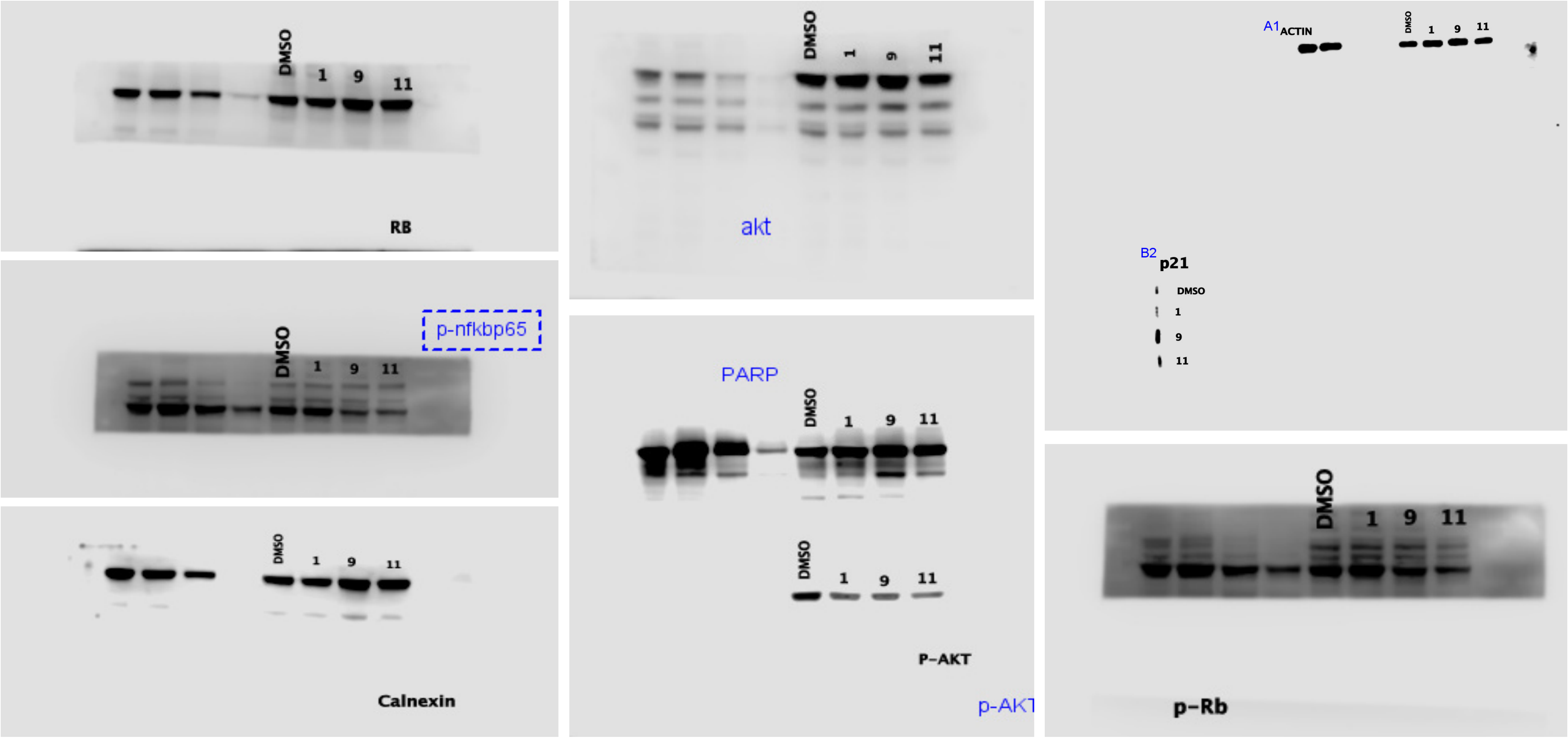

Supplementary Figure S2: Full-length images for western blotting.
